# Explainable deep learning in bloodstain pattern analysis: A pilot study using convolutional neural networks with saliency maps

**DOI:** 10.1016/j.fsisyn.2026.100712

**Published:** 2026-06-27

**Authors:** Vasiliki Pasalidi Chantzi, Jo Millington, Enrico Mariconti, Sherry Nakhaeizadeh

**Affiliations:** a Department of Security and Crime Science, University College London, 35 Tavistock Square, London, WC1H 9EZ, UK, United Kingdom; b SPATTER/ED, Oxford House, 12-20 Oxford Street, Newbury, Berkshire, RG14 1JB, United Kingdom

**Keywords:** Explainable deep learning, Artificial neural networks, Saliency maps, Convolutional neural networks, Bloodstain pattern analysis, Forensic interpretations, White-box BPA XAI methodology

## Abstract

The purpose of this pilot study was to explore the feasibility of applying a novel explainable deep learning (XDL) methodology to classify Bloodstain Pattern Analysis (BPA) patterns. A convolutional neural network (CNN) was applied to classify impact and non-impact BPA patterns. A combination of BPA patterns generated by the researcher were supplemented by open-source BPA datasets and used for the CNN training. This methodology yielded promising results of up to 79% accuracy over 10 folds, validating the feasibility of such research avenues in the field. Furthermore, saliency maps were applied as the CNN's explainability layer, which is a novel application of XDL in BPA interpretations. The study highlights the potential of a novel BPA research stream in XAI while also underscoring the potential of the model to act as a possible reliable alternative tool for BPA experts over manual classification methodologies. This approach does not aim to replace the human element from the forensic science process but rather provide a tool to BPA experts to aid and expedite BPA interpretations without compromising on explainability metrics. It has the potential to increase transparency and trust in deep learning systems, which in turn would increase the reliability of forensic outcomes.

## Highlights

• Explainable deep learning can be used in BPA to help with transparent and reliable interpretations.
• Deep neural networks showed promising results in classifying impact from non-impact bloodstain patterns with accuracies reaching up to 79%.
• Saliency maps proved to be a promising novel methodology to add an explainability layer to deep learning classifications in relation to bloodstain pattern classifications.

## 1 Introduction

Despite significant progress in the field of forensic science [1], the need for more robust, objective and efficient techniques for bloodstain evidence interpretation, classification, and pattern recognition continues to be highlighted as a pressing concern within Bloodstain Pattern Analysis (BPA) [[2], [3], [4], [5], [6], [7], [8], [9], [10], [11]]. Furthermore, challenges persist in relation to differentiating certain bloodstain patterns due to morphological similarities and the lack of diagnostic features distinct for each classification group [7,8,[12], [13], [14]]. Agreeing on a standardised and universally accepted terminology for bloodstains is also an issue of contention among experts and across different countries which has been recognised by Organization of Scientific Area Committees (OSAC) and other researchers in BPA [[15], [16], [17], [18]]. Understanding how bloodstains form, mainly by focussing on their size, shape, and distribution has shown to be important in crime scene analysis and reconstruction [19]. Within forensic science, the use of cutting-edge technology such as machine learning (ML) and deep learning (DL) models has become increasingly applied across numerous classification tasks [20]. Within recent years new innovative approaches to AI have shown the potential of enhancing decision outcomes across numerous forensic domains [21], giving insight into tackling some of the challenges forensic science has faced. Within BPA, some studies have focused on using an automated approach to distinguishing between different types of bloodstains such as impact patterns, spatter distributions caused as a result of gunshot dynamics, and other mechanisms with a varying degree of accuracy [[22], [23], [24]]. Many of these studies have highlighted the need for building less subjective methods allowing for quantitative approaches to aid in pattern classification [25,26]. However, caution has been raised regarding the use of AI models in critical contexts, such as the criminal justice system, due to the fact that the majority of studies conducted to date have relied on using black box approaches. These are arguably unexplainable when used in isolation [[27], [28], [29]]. Legal practitioners, courts and the general public have expressed scepticism over AI evidence used in criminal proceedings which is rooted in the lack of understanding and transparency of how AI systems reach their decisions [30,31]. OSAC's current BPA subcommittee research agenda is also focussing on undertaking white-box studies and establishing a written methodology for BPA classifications [17,18,32,33]; in this regard, explainable AI (XAI) can be seen as a white-box methodology that can therefore contribute directly to OSAC's research needs.

Explainable AI (XAI) refers to artificial intelligence algorithms designed to be transparent and understandable by humans, enabling the interpretation of the internal decision-making logic of the system [34]. With the widespread adoption of AI, there is a growing need for XAI models to address the plethora of existing black-box models, which diminish the level of trust and confidence of end users in their outputs [35]. This urgency stems from the potential risks, errors, and biases associated with AI-enabled decision-making processes. The call for transparency is crucial in the early stages of AI development to avoid the proliferation of biased and harmful systems in the future [36]. XAI plays a pivotal role in unlocking the full potential of AI while acknowledging and comprehending its limitations. By allowing developers to trace the processing steps of a model, XAI enhances transparency and trust in the outcomes, contrasting with the current black box approaches that lack interpretability [37]. In the context of forensic science, such XAI applications are not meant to remove or exclude the human expert from the process but rather assist them in reaching their interpretations [27]. The involvement of a human expert is in fact inseparable and crucial to ensure fairness, transparency, and accountability of AI-enabled forensic interpretations [38].

This paper presents initial proof of concept research that operates at the intersection of XAI and BPA. It leverages the latest advancements in explainable machine learning algorithms to address key challenges in the interpretation of bloodstain pattern evidence, thereby contributing to the ongoing evolution of forensic methodologies and practices. The predominant aim of this research is to showcase how the responsible, ethical and fair use of artificial intelligence within the forensic context of BPA, could be conducted, placing a major emphasis on the transparency of AI decision-making and model explainability.

The primary objective of this paper is to apply novel explainable deep learning (XDL) algorithms for the classification of impact versus non-impact patterns using saliency maps as the explainability layer. This paper is a pilot experimentation that uses deep learning classification models on primarily open-source data and some manually derived BPA data to classify bloodstain patterns and adds an explainability metric to the algorithm using saliency maps. The main binary classification task focussed on impact versus non-impact bloodstain patterns.

The application of a convolutional neural network for binary classification of bloodstain spatter patterns is a novel pursuit, as is the application of saliency maps to the aforementioned model as a means of adding an explainability metric to increase transparency and trust in the model output. The methodology behind generating a pilot deep algorithm model was developed to investigate the feasibility of using open-source data along with manually generated data and deep learning classifiers in BPA, as well as piloting a preliminary XDL model. Ultimately, the goal of this pilot study was to provide insight into the viability of XML applications in BPA classifications and provide feedback to improve and fine tune future planned research in several aspects, including preprocessing of input images, choice of XML algorithms and interpretability and explainability metrics for applied ML algorithms.

## 2 Methodology

The BPA dataset used a combination of data generated manually by the researcher along with two open-source datasets [39,40] amounting to a total of 128 bloodstain patterns. The specific methodologies followed for data collection, data preprocessing and model architecture are outlined below. In total, there were 93 impact patterns and 35 non-impact patterns. In order to ensure a representative and balanced sample, several methodologies were implemented, including stratified sampling and cross-fold validation (see sections 2.3, 2.1.2).

### 2.1 Data collection: open-source datasets

A limited number of researcher-generated samples was supplemented with open-source datasets in order to pilot the algorithm model. Currently, two high-quality open-source impact pattern datasets are available for training and educational purposes: one for impact patterns generated as a result of beating activities [40] and another for impact patterns developed from gunshot backspatter [39]. An overall description of the dataset is provided with full details being available from the open-source publications [39,40].

#### 2.1.1 Beating impact dataset

The beating impact pattern dataset comprises a total of 61 images of spatters presented on white targets with all being subsequently scanned to produce high quality images of varying sizes. For most spatter patterns, around 1 mL (ml) of blood was used to develop a pool of blood on which striking devices impacted. Two setups were used to mimic beating activities: one was a hockey-puck rig which involved blood on a flat surface being hit by a cylindrical rod with different forces; and the other was a cylinder rig where blood was squeezed between the surfaces of two flat cylinders. The main parameters measured for each spatter pattern were the impact velocities, which ranged from 2 m/s to 8 m/s, and the distance between the target and the blood source, which ranged from 30 cm to 2 m. Most of the patterns were a result of single impacts with few exceptions involving multiple impacts from different horizontal and vertical distances. All 61 images were used for this pilot study.

#### 2.1.2 Gunshot impact dataset

The gunshot impact pattern dataset comprises a total of 68 high quality images of varying sizes showing spatters presented on white targets. The amount of blood used to soak a sponge or closed cavity for the experiments ranged from 2.5 ml to 175 ml of blood which were impacted with a bullet. Three main bullet types were investigated: ‘flat’, ‘pointy’, and ‘round’ and their velocity ranged from 285 m/s to 987 m/s. The majority of the spatter generated across the repetitions was captured on vertical targets, although a few horizontal targets were produced. The distance between the vertical targets and the blood source ranged from 30 cm to 120 cm. For most repetitions, the interactions of muzzle gas and spatter were minimised by the projectile first passing through a vertical target and a few targets were produced where the muzzle gases were permitted to interact with the backspatter.

In order to set up a baseline algorithm, only patterns that reflected strict controlled conditions were included. Each image was therefore assessed manually to determine the suitability of the patterns. Patterns that were split over two images or targets that had very little spatter on them were excluded (to avoid partial pattern capture), as well as targets that had visible black markings on them (to minimise gunshot artefact). This triage process resulted in a total of 32 images being used from this dataset.

### 2.2 Data collection: manually generated patterns

35 patterns were generated in collaboration with SPATTER/ED [41] using defibrinated horse blood [42]. The non-impact dataset comprised of 18 projected patterns and 17 secondary spatter patterns i.e. comprising satellite stains produced from drip patterns. In order to increase the ecological validity of the study, a small number of patterns that presented artifacts, e.g. having a scale on them, were retained in the dataset.

#### 2.2.1 Secondary spatter pattern procedure

**Fig. 1:**
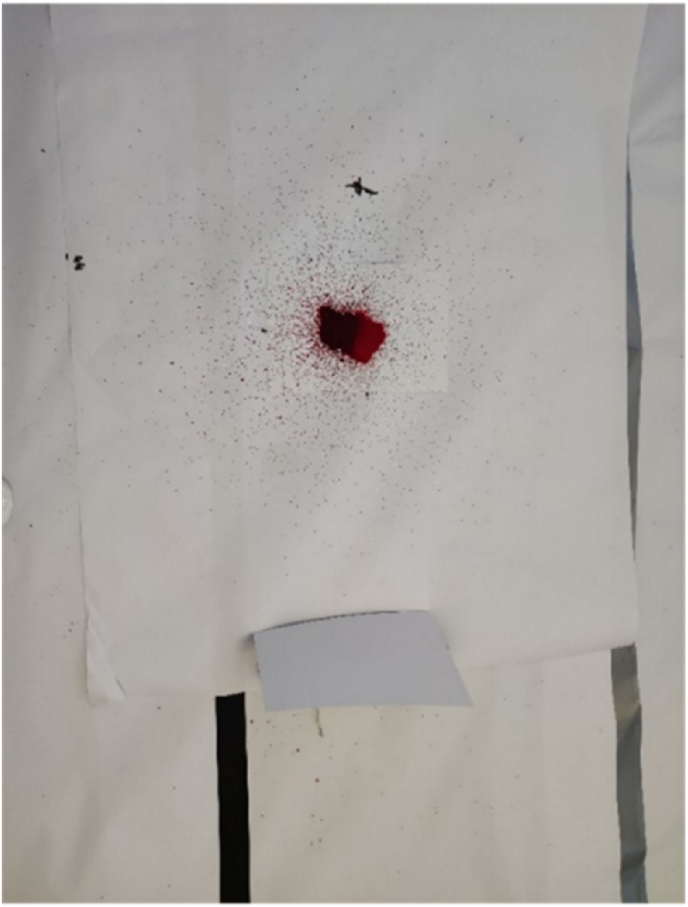
The setup relating to the target placed at the 50 cm location.

**Fig. 2:**
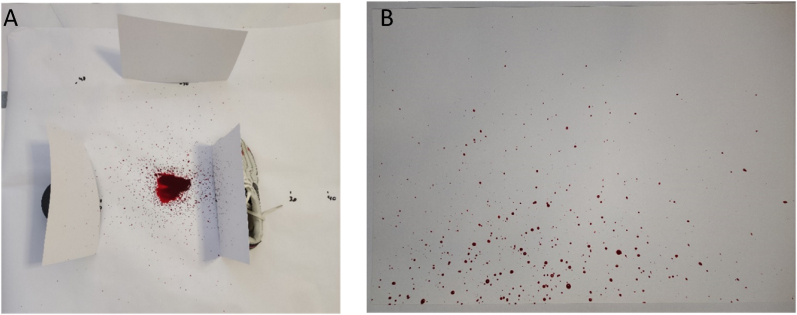
Panel A shows the three locations where targets were placed 10 cm, 20 cm and 30 cm away from the origin spot in order to capture secondary spatter. Panel B shows the resulting spatter on the target located at 10 cm.

**Fig. 3:**
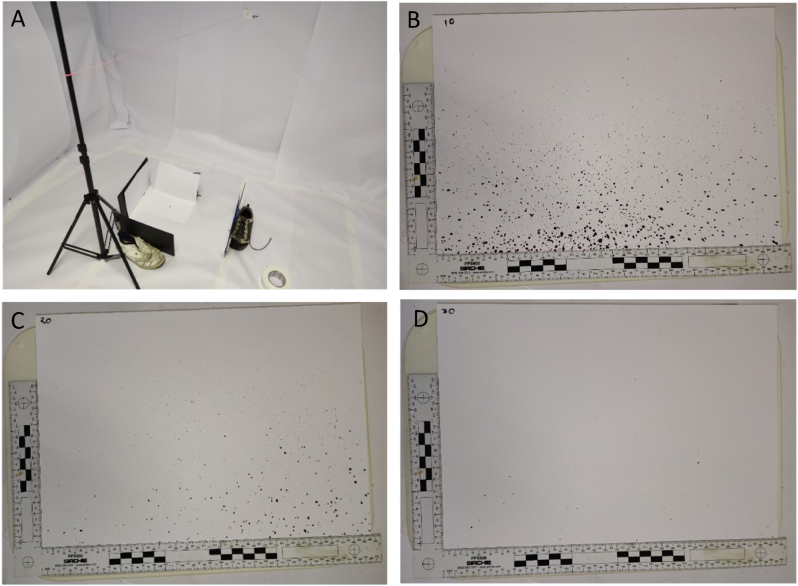
Panel A shows the tripod setup with a string attached to it and the wall at a height of 100 cm from which blood was dripped on to the origin spot. Panels B-D show the results of one replication of this methodology where 32 ml of blood were dripped at a fast rate 10 cm, 20 cm and 30 cm away from the origin spot respectively.

A central location (the origin) was marked on a horizontal white target. Vertical white targets were first placed at linear increments of 10 cm up to 50 cm and at 100 cm away from the origin (Fig. 1), and subsequently in a box arrangement around the origin at 10 cm, 20 cm, 30 cm, and 40 cm distances from the origin (Fig. 2). Gravitational blood droplets were then dispensed repeatedly from a height of approximately 100 cm onto the origin location and the resulting satellite stains captured onto the placed targets. Two qualitative drip rates were employed, fast and slow. ‘Slow’ was measured as dropping one blood drop per second from a disposable pipette into the blood pool. ‘Fast’ involved ejecting the whole volume from the pipette. Each process was repeated 6 times with the ‘fast dripping’ mode using a total of 32 ml of blood. A total of 17 targets each containing variable amounts of spatter were created during this exercise (Fig. 3).

#### 2.2.2 Projected spatter patterns procedure

**Fig. 4:**
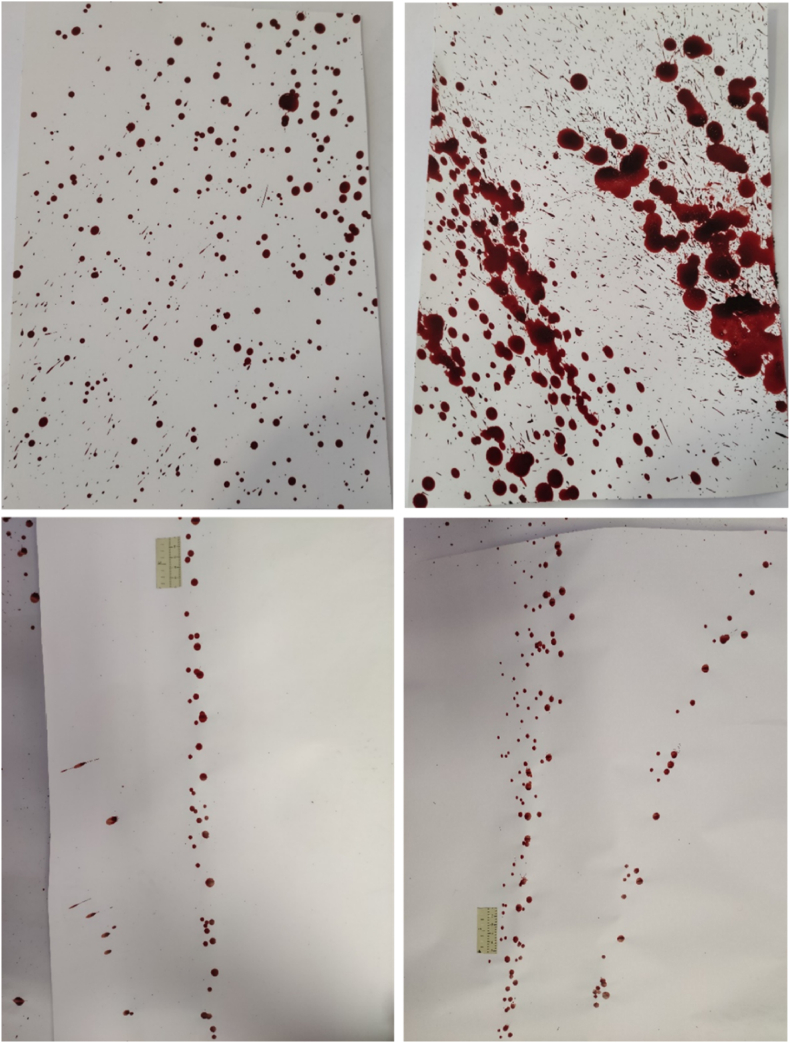
Selection of projected bloodstain patterns collected during SPATTER/ED's training courses.

Due to the complexity of fluid dynamics and anatomy involved in generating this type of pattern, accurately replicating realistic projected pattern types poses considerable challenges. Simulated projected bloodstain patterns were therefore created using SPATTER/ED's ‘arterial pump’ [43]. This machine facilitates the projection of blood under variable pressures, simulated ‘heart rate’ and damaged vessels. White targets were positioned vertically and horizontally within the experimental space to capture multiple projections of blood. Simulations of arterial spurt, rain and gush patterns were collected, and in some cases, where droplets impacted others at the target surface, were associated with pronounced satellite stains. A total of 18 targets each containing variable amounts of spatter were created during this exercise (examples in Fig. 4).

### 2.3 Data pre-processing and standardisation

Prior to training each deep learning model, a layer was added to standardise all BPA images to a uniform dimension as is standard procedure when dealing with CNNs [44]. Initially, the images were all converted to 512 × 512 pixels due to scalability and throughput concerns i.e. how fast the images can be processed by the models, and to speed up the algorithm, the images were reduced to 256 × 256 pixels. The processed dataset comprised a total of 128 BPA images, each labelled with corresponding class identifiers, of which 93 were derived from the open-source dataset described above, and 35 manually generated non-impact patterns. The dataset was split into training (70%), validation (15%), and testing (15%) sets. The distribution of classes within the dataset was found to be well-balanced, ensuring that the model was exposed to a representative range of BPA patterns during the training process.

### 2.4 Model architecture and DL algorithm methodologies

#### 2.4.1 Deep learning classification algorithm

A convolutional neural network (CNN) architecture was chosen for this research as the input data consist of images and CNNs have proven efficacy in image classification tasks and computer vision tasks [45,46]. The experiments were conducted on a Google Colab workbook equipped with a T4 GPU and ran on Python 3.10 and TensorFlow 2.15 using the high RAM allocation.

**Fig. 5:**
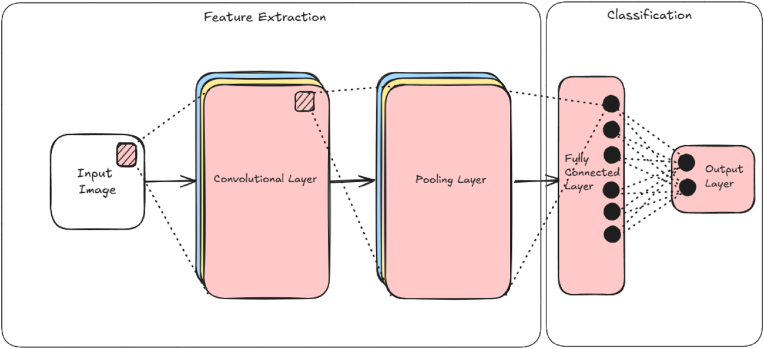
CNN model architecture that was used for binary classification in this research, showing the interactions between each layer during feature extraction and classification functions. Created using Excalidraw.

Each CNN model consisted of multiple layers, including dense, convolutional, and max pooling layers, with associated batch normalisation, pooling and shape sizes accordingly. Each layer of the CNN serves different purposes: the convolutional layer performs feature extraction by applying filters to the input images; the pooling layer is also part of the feature extraction by reducing the dimensionality of the image and computation load needed, while the fully connected layer is responsible for performing the classifier prediction [45]. An example of the architecture of a CNN model is provided in Fig. 5. In terms of training the model, test, training and validation sets were used. In order to address the small number of input data and to prevent over-fitting, both a validation set was included, and k-fold cross-validation was performed for each model fitting. The process for k-fold cross-validation used was to firstly split the dataset into k number of folds and for each training split using a different fold as the train/validation set [47].

The binary cross-entropy loss function was selected for its suitability in binary classification tasks [48]. It effectively measured the dissimilarity between the predicted probability distribution and the true label. The Adam optimization algorithm was utilized to train the model, owing to its adaptive learning rate and momentum properties, which facilitated faster convergence and improved training efficiency. To address the small number of data and the size imbalance between the two classes, a 10-fold cross-validation strategy was employed to ensure the robustness of the model using stratified splitting. This meant that the training and validation process was repeated 10 times, each time using a different subset as the test/validation set. That is, the total dataset was first stratified split for 10 folds, and each fold had a separate training, test and validation set. For each fold, the model was fit using the training and validation set whereby the training accuracy, training loss, validation accuracy and validation loss were recorded and plotted in graphs (see Results). Each model was then evaluated against both the test and validation sets and a final prediction was made against the test data that were used for the calculation of the classification metrics table (see Results, Table 1).

**Table 1:** The output of the classification report of the model training, showing the accuracy, precision, recall and f1 score for each fold on the test dataset.

Fold	Accuracy	Precision	Recall	F1 Score
*1*	68%	79%	79%	79%
*2*	63%	71%	86%	77%
*3*	*47% (min)*	*64% (min)*	*64% (min)*	*64% (min)*
*4*	**79% (max)**	81%	**93% (max)**	**87% (max)**
*5*	74%	80%	86%	83%
*6*	58%	71%	71%	71%
*7*	**79% (max)**	**86% (max)**	86%	86%
*8*	74%	80%	86%	83%
*9*	63%	73%	79%	76%
*10*	68%	79%	79%	79%
*Average*	**67.3%**	**76.4%**	**80.9%**	**78.5%**

#### 2.4.2 Explainable deep learning algorithm

Regarding XML, the primary method used were saliency maps which are created by ‘tracking’ deep learning models in order to identify which pixels in an image were more ‘salient’ in the training of a model i.e. on which pixels the model concentrated most when calculating its output. Saliency maps are a specific example of an explainability technique used in the context of deep learning models, particularly in the field of computer vision [49]. These maps highlight the most relevant features or pixels in an input image that contribute to the model's prediction [50,51]. By visualizing the areas of the input that the model focuses on, saliency maps provide valuable insights into how the model processes and analyses the input data, thus enhancing the interpretability of the model's decision-making process. The methodology used for this research was based primarily on Rizwan's work [52] using the gradient tape with some slight adaptations. By applying saliency maps, it was possible for the algorithm to conduct a side-by-side comparison between the maps and the original bloodstain pattern and thereby identify which pixels were the most salient for each algorithm applied.

## 3 Results

### 3.1 Impact versus non-impact binary classification

The training process, as detailed above, resulted in a convergence of the model within 15 epochs, with the training loss decreasing steadily and the validation loss stabilizing, indicating that the model did not overfit the training data i.e. it did not fit too much on the training data and thus have lower accuracy in validation and test data. The performance of the model was evaluated using standard classification metrics, including accuracy, precision, recall, and F1 score (Table 1).

In this instance, the trained model achieved an average accuracy of 67.3% and reached a maximum accuracy of 79%, demonstrating its robust capability in accurately classifying BPA images. Across folds there were some discrepancies in the metric values, predominantly for accuracy; however outside of some outliers, the model seems to perform reasonably well and the values for each metric seem to remain relatively consistent throughout all 10 folds. The accuracy ranged from 47% to 79% across different folds, indicating some variability in the model's performance, even though solely considering accuracy may not be the best metric for imbalanced datasets. The precision ranged from 64% to 86%, showing some variation in the model's ability to make precise correct predictions across different folds. The recall values ranged from 64% to 93%, showing some variability in the model's ability to capture positive instances across different folds. Lastly, the F1 score ranged from 64% to 87% showing more consistency across the folds. More specifically for the impact vs non-impact binary classification task, the output of these metrics for each fold is summarised in Table 1.

**Fig. 6:**
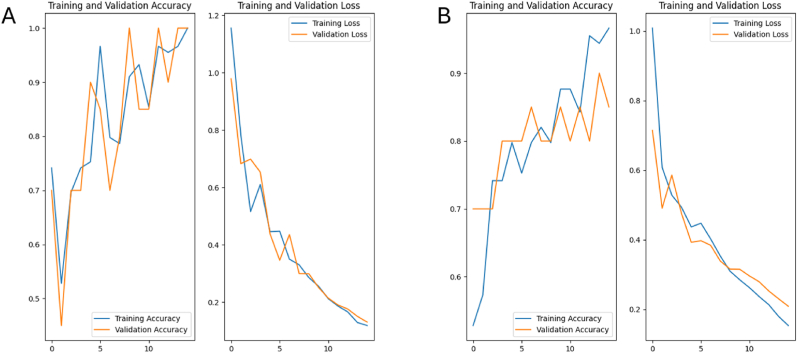
Graphs A and B show the training and validation accuracy and training and validation loss for fold 3 and 4 respectively as an example of the trend they follow as the epoch iterations increase during the model training. In the beginning both training and validation accuracies are low but increase with increased iterations, exhibiting a sigmoidal curve. In contrast, the training and validation loss decrease.

Furthermore, for each fold a graph was created plotting the training and validation accuracy and training and validation loss across the epochs (see Fig. 6). By plotting these four graphs for each fold, the performance of the model can be measured, including whether the model may be overfitting. In an ideal scenario, it would be expected that both training and validation accuracy increase after each epoch and both accuracies to be within the same range. If a model produces much higher training accuracy than validation accuracy, then that could be a likely indicator that the model is overfitting the data to the training dataset. In this experiment, all graphs show that training and validation accuracies are low in the first epochs as would be expected, both graphs seem to increase and stabilise over the remaining epochs without much deviation from one another. Similarly for the training and validation losses, it would be expected that the more epochs a model is being trained, the training and validation losses will tend to be minimised as much as possible. In this experiment it is shown that graphs for the training and validation losses follow the expected curve, declining significantly over the course of the training epochs with both lines remaining in a similar range. All folds exhibited the same trend, including the worst and best performing folds shown in Fig. 6. Therefore, these graphs are supportive of the scenario that the model does not show signs of overfitting or underfitting. However, despite no clear signs of overfitting, considering the metric outcomes shown in Tables 1 and it is possible that allowing more time during the training could have benefitted model fitting.

### 3.2 Saliency maps as an explainability metric

Results from a saliency map analysis on a binary classification model provide insights into the features or regions of the bloodstain pattern images that the model considers most important for making its predictions. Saliency maps therefore highlight the feature importance of the areas of the input data that contribute the most to the model's decision-making process. A visual representation of the relevance of different pixels of the bloodstain patterns is shown as bright regions. These correspond to pixels in the pattern image on which the model relies for its classification tasks, while darker regions are less relevant for the model.

**Fig. 7:**
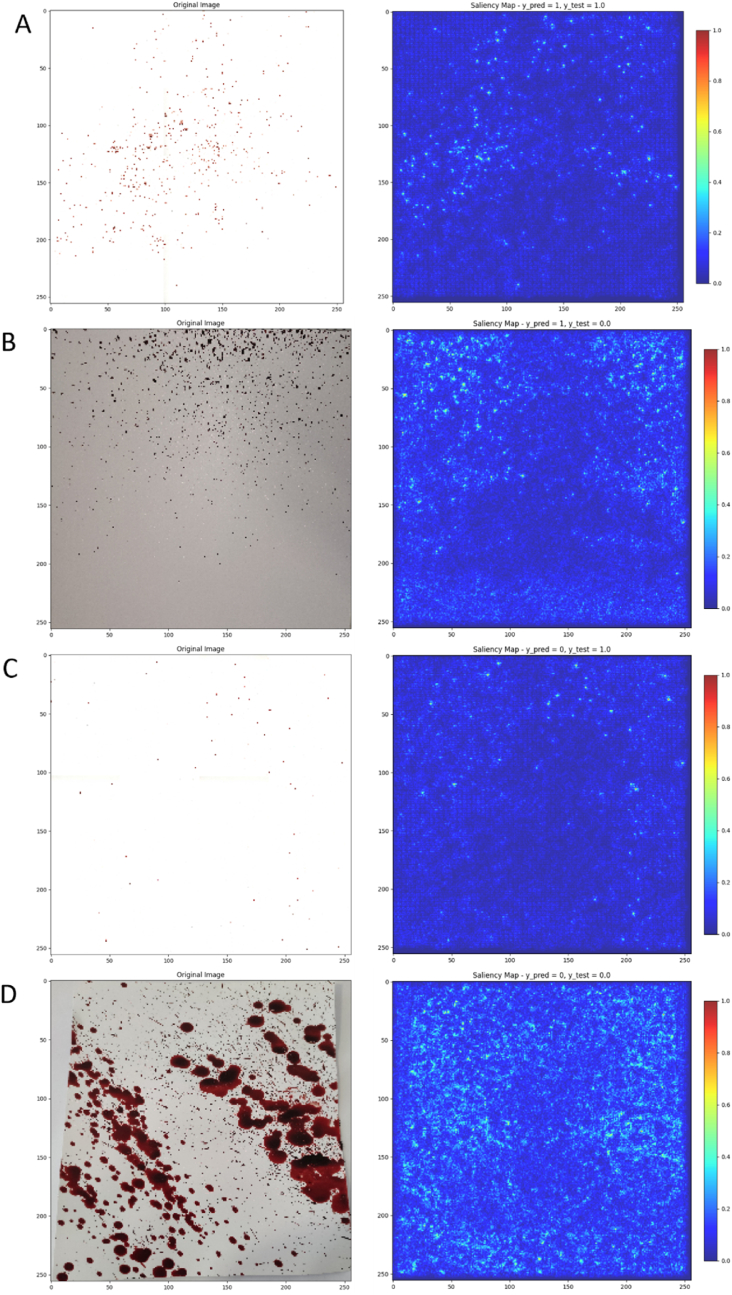
Saliency maps of models showing prioritisation of spatter features in the bloodstain pattern images. All panels show the original image fed into the machine on the left side and the saliency map corresponding to classification of the respective image on the right side. y_pred and y_test are the values of the predicted and actual classification labels of the test data, with 1 corresponding to impact patterns and 0 to non-impact patterns. Panel A shows the saliency map for an impact pattern that was correctly predicted by the model, while panel B shows the saliency map for a secondary spatter patter that was incorrectly predicted. Panel C shows a secondary pattern incorrectly classified as an impact. Panel D shows a projected pattern correctly classified as a non-impact pattern.

For the purposes of this experiment, saliency maps were manually evaluated based on the BPA domain knowledge focussing on whether the model focussed on spatters or absence thereof, both of which can be insightful in BPA, and whether the prediction of the model was correct. For every fold, the saliency map for each image in the test dataset was created along with the input test image. The model prediction for that image was also recorded. There were 19 images in total in the test dataset for each fold. Overall, for the most part, when the saliency maps showed that the model was focussing predominantly on spatters in the test image, the prediction of the model tended to be correct; while when the model focused on non-spatter areas of the patterns, the prediction tended to be incorrect. For instance, Fig. 7 A shows the saliency map for an impact pattern, where the model seems to prioritise and focus predominantly on specific spatters across the pattern and correctly predicting it as an impact pattern. There were some occasions where saliency maps of non-impact patterns were showing that the features of the spatters in the image were prioritised yet elicited an incorrect prediction (Fig. 7 B). This was the case predominantly for ‘secondary spatter’ patterns whose differentiation from impact patterns remains challenging in BPA [7,8,53]. Interestingly, there were also instances where saliency maps which showed that the model was focussing on spatters on the images incorrectly, classified non-impact patterns as impact (Fig. 7C). This phenomenon was predominantly manifested for impact patterns of targets that were placed at greater distances from the origin i.e. with less dense and more spread-out spatter stains. It is the authors' experience that, when spatter stains at the periphery of an impact pattern, especially when viewed in isolation or in the context of partial pattern capture, may not align with all of the objective criteria for impact pattern classification, and may also align closely with the features of satellite spatter from a drip pattern. This highlights the requirement for further modelling using patterns that are created by different mechanisms that show morphological similarities. This seemed to be less of an issue with non-secondary non-impact spatter patterns such as projected bloodstain patterns, where the difference between their features and that of impact patterns seemed to be picked up by the model as shown in the saliency map and correct prediction of the test projected pattern in Fig. 7 D.

**Fig. 8:**
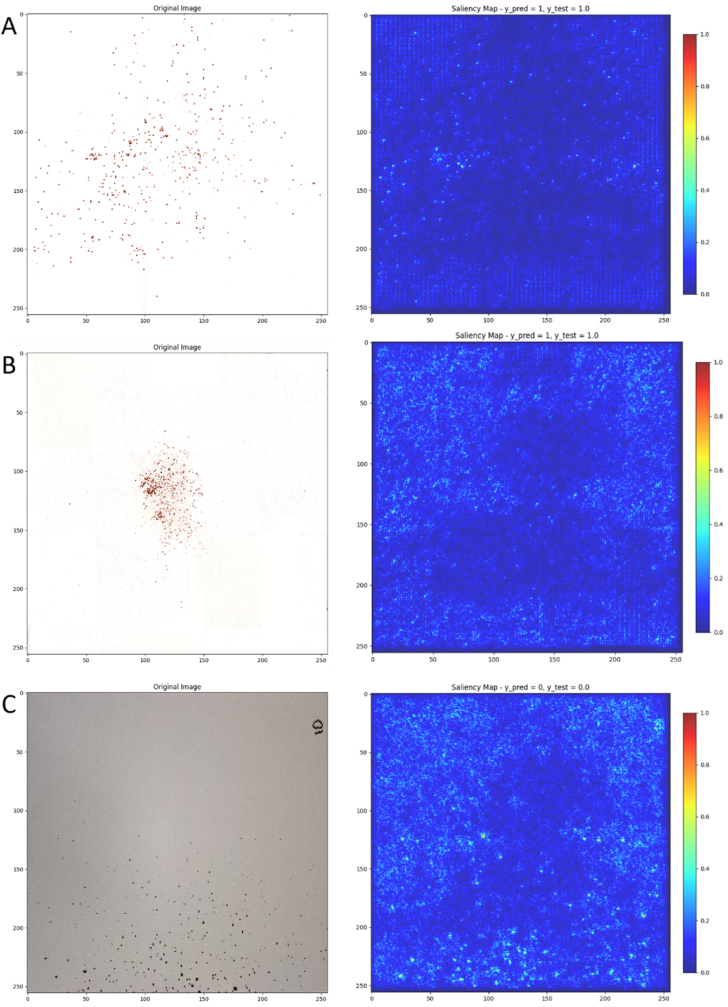
Saliency maps of impact spatter patterns (A-B) and a secondary pattern (C). All panels show the original image fed into the machine on the left side and the saliency map corresponding to classification of the respective image on the right side.

Interestingly, there were some instances where the saliency map indicated that the model was focussing on pixels outside of spatters, either by ignoring spatters in the pattern or by focussing on image artifacts. Fig. 8 A shows the saliency map of an impact bloodstain pattern that focused on some partial spatters with a void in the middle of the pattern yet predicted the instance correctly. In contrast to that, Fig. 8 B shows the saliency map of another impact pattern that shows a void where the pattern is and instead shows a priority around the pattern. This is likely due to artifacts in the quality of the picture, whereby the model is trying to extract information from the digital data sources instead of the impact pattern itself. In this case, the model technically correctly predicted the pattern's classification, yet the saliency map provides an extra layer of explainability and way to improve the model. This suggests that the decision-making of the model needs further fine tuning. Similarly Fig. 8C shows the saliency map of a correctly predicted ‘secondary’ pattern for which the model considered both the spatters and background image artifacts. This suggests that more data are needed in order to have a balanced dataset for both categories in order to further fine tune the model.

## 4 Discussion

Overall, this pilot study demonstrates that saliency maps can effectively visualise the feature priorities of a CNN during each fold of training, providing an important layer of explainability for BPA classification tasks. As the first application of XAI saliency-based methods in BPA, this work offers a promising, novel direction for developing transparent, white-box analytical approaches aligned with OSAC's current research priorities [17,18,32,33]. This can have a considerably positive impact on BPA classifications and pave the way forward for a new stream of XAI research within BPA. The CNN achieved average classification metrics of approximately 70–80% (Table 1), indicating a promising baseline model. The saliency outputs provided an explainability layer into the model's intrinsic decision-making and further revealed which image features influenced its predictions, highlighting both strengths and areas requiring refinement. Although the saliency map evaluation in this study was primarily visual, the results indicate that further fine-tuning is necessary, particularly to incorporate additional parameters that could help the model better distinguish between impact and non-impact patterns. For instance, it appears that the model, despite focussing on the spatter features could not always correctly differentiate between impact versus non-impact patterns (Fig. 7B and C). Building upon this research, a future extension would be to incorporate current analyst error rates, however, considering that this is a pilot study, it would be an overreach to use the current model for such comparisons [9,15,53].

Future versions of the model should also improve its ability to identify positive and negative visual contexts in an image, such as distinguishing meaningful pattern voids from irrelevant image noise. Achieving this will require larger datasets and more comprehensive definition and standardisation of image-capture parameters, including lighting, resolution, and signal-to-noise characteristics [50], to support high ecological validity and be of universal use. It is possible, however, that the saliency maps were focussing on spatter-adjacent features or other implicit spatter density features in those cases were it seemed to focus on perceived artifacts. For instance, it is possible the algorithm was considering the distance between different spatters or dispersion of stains as well as void areas in the same pattern in order to derive this as a feature. Additional work is also needed to improve the objectivity and automation of saliency-map evaluation. Because the present study used linear approximation methods, the resulting maps may be limited in accuracy. Incorporating non-linear approximation techniques recommended in the literature [49,54] would allow for better comparison and improved interpretability.

Explainability in the context of machine learning refers to the ability to explain and interpret the decisions made by machine learning models in a manner that is understandable to humans [55]. It involves providing meaningful explanations for the predictions or outcomes generated by these models, thereby making the decision-making process transparent and comprehensible [56]. This is particularly important as it enables users to trust and rely on the outputs of these models, especially in high-stakes domains such as forensic science where legal outcomes can have significant and life-altering implications [57,58]. The need for explainability arises from the increasing use of complex, black-box machine learning models, such as deep neural networks, whose internal workings are often difficult to comprehend [[59], [60], [61]]. As a result, there is a growing demand for methods and techniques that can elucidate the rationale of why models reach particular predictions [[62], [63], [64], [65]]. Explainability is thus crucial for ensuring accountability, transparency, and fairness in machine learning systems, as it allows stakeholders to identify and rectify any potential biases or errors present in the models, while also informing algorithm refinement [62,66,67]. Within this context, this pilot study demonstrates how XAI-based approaches can begin to address long-standing concerns around the transparency of BPA classification and interpretations.

Despite the widespread adoption of saliency maps in XAI domains, there remain several issues with their interpretations as their methodology is not always transparent nor systematic, this in turn may limit their reliability [68]. Moreover, the absence of established evaluation metrics and known error rates, coupled with the potential for noise within saliency outputs may render their interpretation more complicated [69]. Improvements such as incorporating rectified gradients or addressing input-bias effects have been proposed to enhance clarity and interpretability [70]. Applying such modified techniques within BPA to build upon the research stream proposed in this study would represent a meaningful contribution to XAI research in BPA [71,72].

The visualisation of the relevance of each feature/pixel can provide considerable insight both in terms of ‘positive’ relevance, i.e. saliency maps that concentrate on specific individual or grouped spatter stains, as well as ‘negative’ context, i.e. saliency maps where the model focuses more on the absence of spatter stains or patterns. These dual aspects can reveal potential model biases and guide targeted improvements. Given the dynamic and variable nature of BPA pattern formation, saliency maps could support expert analysts by enabling superimposed or side-by-side comparisons between original images and the model's focal regions, as shown in Fig. 7, Fig. 8, thereby helping assess the reliability of model outputs. Strong and consistent saliency maps across multiple spatter patterns of the same classification, in this case impact versus non-impact patterns, would be indicative of greater confidence and robustness in the model's decision-making and performance, whereas highly variable maps would likely signal greater uncertainty in the model's predictions. Having a larger dataset and more representative, balanced sample sizes for each classification category could help mitigate higher variability in the saliency maps of the CNN model.

## 5 Limitations

The main limitation of this study was the restricted amount of data available for machine learning. Furthermore, there was a disproportionate number of images for each label group with only 35 of the 128 images representing the non-impact category, creating class imbalance and affecting model performance, particularly during early training. To address the small dataset size in this pilot study, a hybrid validation strategy was used (10-fold cross-validation alongside a test set). Although this is not standard practice, this approach mitigated some of the challenges to an extent. The need for high-quality image data should be stressed as it is possible that the reduction in pixels of the input images adversely affected the model performance. The dataset combined manually generated photographs, captured on a standard mobile device, with two open-source datasets, each produced under different experimental conditions. These variations in image quality, resolution, lighting, and methodology introduce inconsistencies that are especially consequential in computer-vision tasks. Additionally, the open-source datasets lacked uniform image dimensions and contained insufficient repetitions across key experimental variables (e.g., distance from blood source, impact mechanism, bullet type). Because of the limited sample size, all impact patterns, whether generated through beating or gunshot mechanisms, were merged into a single class, despite methodological differences that could influence stain morphology. Whilst open-source datasets can be very beneficial to address limited sample sizes, they do not come without limitations. The main consideration, as outlined above, was that both datasets comprised impact patterns only. Furthermore, the total number of images remains incredibly small and considering that multiple parameters were investigated, including for instance the distance between targets and blood source, different beating mechanisms and different bullet types, very little repetitions were made for each condition and each combination of parameters. This can have considerable impact on machine learning algorithms as there are not enough data for each parameter in order to be able to accurately establish a baseline. In the case of gunshot backspatter patterns, different targets were placed next to each other to make one target following the generation of the impact, leading to noticeable gaps in pixels that the algorithms could pick up.

Similar limitations affected the non-impact class. Due to the difficulty of generating certain non-impact patterns, projected and secondary patterns were combined into one category. Although binary classification requires two clearly defined groups, due to the class imbalance it would not be representative to pursue a multi-class classification avenue at this stage. Future work should therefore consider testing such alternative algorithms, increasing sample sizes, and incorporating data augmentation to counterbalance dataset constraints. These issues highlight the broader challenge of acquiring a wide range of high-quality fit-for-purpose experimental and real-world BPA datasets within forensic research, which include patterns formed on varied surfaces and under differing environmental conditions, in order to increase ecological validity.

Computational constraints also influenced model development. Despite using the highest-tier Google Colab subscription, the system experienced slow performance and occasional crashes, necessitating a reduced learning rate and fewer epochs per fold. Although the models did not show obvious signs of overfitting, the modest accuracy levels suggest that extended training using more epochs, different learning rates, and more computational resources may have improved performance. Further fine-tuning of model architecture and hyperparameters is therefore warranted. Because target surface properties influence blood behaviour, future models may benefit from incorporating variables such as substrate type, absorption, and texture and other blood-target interactions to help distinguish between different types of impact and non-impact mechanisms.

Future research should pursue larger and more diverse datasets and consider advanced deep learning architectures or transfer-learning approaches to improve generalisability in real-world forensic settings. Incorporating data from varied surfaces and colour backgrounds would more accurately reflect practical BPA challenges, where detecting and visualizing stains is often a prerequisite to analysis. Subsequent studies should explore either a dedicated hold-out test set or a purely cross-validated approach to strengthen methodological rigour.

## 6 Ethical ramifications

Since this is a novel study on XAI applications in BPA, it is important to note that there are also ethical ramifications of using AI tools in BPA and forensic science decision-making which extend beyond pragmatic and methodological questions as they tend to be deeper, complex and even ontological in nature. Even though the concept of AI and XAI are not new [73], the rapid technological advancements of the past decades have made AI a ubiquitous and undeniable reality of human life, whereby AI is an interwoven and indispensable facet of humanity [74].

Whilst XAI, including as employed in this paper, is not meant to replace the human expert from the forensic science process, it is the author's aim to validate XAI so that it can act as an intelligent aide for experts when reaching their BPA interpretations.

Former Chief Business Officer at Google X, Mo Gawdat, has highlighted that the undeniable reality of the universal adoption of AI means that there is a time-sensitive need for human actors to readily and proactively engage in conversations and implementations of ethical and responsible AI use before the exponential growth of AI renders any such attempts futile in the future and outpace efforts to regulate it [75].

Perhaps the two most alarming consequences of readily available easy to use AI algorithms are the potential for them to be misused, intentionally or unintentionally, by maleficent actors, or that such algorithms can operate in a non-objective manner: either due to biased, incomplete, or otherwise flawed training data, or due to insufficient transparency regarding how decisions are generated [76].

Therefore, the ethical implications of using AI in forensic science are multifaceted and require a comprehensive framework that addresses the concerns of various stakeholders, ensures transparency, accountability, and fairness, and considers the cultural context in which AI systems are deployed [77,78]. The diverse set of stakeholders involved in issuing AI principles and policies reflects the strong interest in shaping the ethics of AI to meet their respective priorities [79]. However, there are concerns that the current approach to AI ethics is largely ineffective and prone to manipulation, particularly by industry actors [80].

In fields such as healthcare and surgery, there is a pressing need for further research to investigate ethical challenges associated with AI adoption related to human agency, accountability for errors, privacy, transparency, and fairness, underscoring the complexity of ethical issues in integrating AI into medical practices [81,82]. Similar issues arise in forensic science, where ethical practice is essential for maintaining the reliability and credibility of expert evidence [27,83]. Furthermore, the portrayal of ethical issues of AI in the media has captured the hopes and fears arising from the rapid introduction of AI technology into society, reflecting the broader societal impact of AI ethics [84]. The growing emphasis on embedding ethical considerations directly into the development of AI tools, particularly in medical contexts, reflects increasing recognition of the need for responsible integration and AI oversight [85]. Cultural factors additionally shape how ethical concerns are interpreted and prioritised, emphasising the nuance and importance of contextualised ethical frameworks [86]. Future planned studies will therefore take the ethical aspect of AI into consideration when generating data, training the dataset, as well as creating the final model to ensure a seamless AI integration BPA applications for interpretation purposes.

## 7 Conclusion

The initial results of the experimental analysis highlight the potential effectiveness of the developed deep learning classification algorithm in accurately identifying and classifying impact versus non-impact patterns. Even though there is room for development and a requirement to expend the scope of the datasets, the relatively high accuracy achieved by the model underscores its potential as a possible tool to be further developed for forensic investigators and analysts, offering an efficient alternative and arguably significant improvement over traditional manual classification methods. By automating the classification process, the model has the potential to increase transparency, throughput, support evidence-based bloodstain classification and reduce the risk of subjective interpretations of pattern classifications. Overall, the experimental findings show the efficacy of the developed explainable deep learning classification algorithm in BPA analysis and classification, underscoring its potential to significantly streamline and expedite the forensic investigation process. The application of both a CNN and an explainability layer, particularly that of saliency maps in the classification of bloodstain patterns, are two novel pursuits in this field as it applies to BPA pattern interpretations.

The findings of this initial research are hoped to pave a new stream of the application of XAI research for BPA interpretations and lay a baseline avenue for contributing to the use of technological and transparent models to the advancement of BPA classification methodologies. With further research, BPA analysts would only stand to benefit from a powerful, transparent and explainable tool for accurate and efficient evidence classification. Additionally, this initial study offers valuable insights and raises further questions into the practical applications of AI in forensic science, paving the way for further advancements and innovations in the domain of forensic analysis and investigation techniques.

## 8 Declaration of generative AI and AI-assisted technologies in the manuscript preparation process

During the preparation of this work the author(s) used generative AI tools, including scite. ai, Copilot in Word and ChatGPT, to assist in developing an initial structural template for the literature review and to refine wording for clarity and conciseness. After using these tools, the author(s) reviewed and edited the content as needed and take(s) full responsibility for the content of the published article.

## CRediT authorship contribution statement

**Vasiliki Pasalidi Chantzi:** Conceptualization, Formal analysis, Investigation, Methodology, Resources, Software, Validation, Visualization, Writing – original draft. **Jo Millington:** Methodology, Resources, Supervision, Writing – review & editing. **Enrico Mariconti:** Supervision, Writing – review & editing. **Sherry Nakhaeizadeh:** Supervision, Writing – review & editing.

## Declaration of competing interest

The authors declare that they have no known competing financial interests or personal relationships that could have appeared to influence the work reported in this paper.
